# Anti‐Inflammatory Effects of Spexin on Acetic Acid‑Induced Colitis in Rats via Modulating the NF‐κB/NLRP3 Inflammasome Pathway

**DOI:** 10.1002/jbt.70285

**Published:** 2025-05-05

**Authors:** Sevil Arabacı Tamer, Fadime Köse, Sevinç Yanar, Özcan Budak, Cahit Bağcı

**Affiliations:** ^1^ Department of Physiology School of Medicine, Sakarya University Sakarya Türkiye; ^2^ Department of Histology and Embryology School of Medicine, Sakarya University Sakarya Türkiye

**Keywords:** inflammation, NLRP3, oxidative damage, spexin, ulcerative colitis

## Abstract

Ulcerative colitis is a chronic inflammatory bowel disease characterized by inflammation and ulcers in the lining of the colon and rectum. Spexin is a novel peptide with antioxidant and anti‐inflammatory properties. This study aims to elucidate the therapeutic effects and underlying mechanisms of spexin in mitigating acetic acid‐induced colitis in rats. Male Sprague Dawley rats were assigned to control (*n* = 14) and colitis (*n* = 21) groups. Colitis was induced via 5% acetic acid (AA) administration (1 mL, intrarect). Post‐induction, rats received subcutaneous saline (1 mL/kg), spexin (50 µg/kg/day), or oral sulfasalazine (500 mg/kg) for 5 days. Control groups received saline or spexin. After 24 h of the final treatment, colons were evaluated macroscopically, and levels of tumor necrosis factor (TNF)‐α, interleukin (IL)‐1β, IL‐18 were determined by ELISA, oxidative stress markers myeloperoxidase (MPO), malondialdehyde (MDA) and glutathione (GSH) levels were measured spectrophotometrically and NOD‐like receptor pyrin domain‐containing 3 (NLRP3), nuclear factor‐κB (NF‐κB), caspase‐1 proteins were analyzed with *Western Blot* alongside histopathological assessments. Colitis induction significantly elevated macroscopic damage scores, stool consistency, inflammatory cytokines, MDA, MPO, and NLRP3, NF‐κB, caspase‐1, while reducing GSH levels (*p* < 0.001–0.01). Microscopic evaluations confirmed increased necrosis, submucosal edema, and inflammatory cell infiltration (*p* < 0.001). Spexin reversed these effects by enhancing GSH levels (*p* < 0.01), reducing macroscopic/microscopic scores, cytokines, MDA, and MPO levels (*p* < 0.05–0.001), and suppressing NLRP3, NF‐κB, and caspase‐1 activation (*p* < 0.01–0.001). For the first time that spexin ameluates acetic acid‐induced colitis in rats by modulating the NF‐κB/NLRP3 signaling pathway, reducing oxidative damage, enhancing antioxidant capacity, and suppressing inflammation.

## Graphical abstract

Spexin exhibits a therapeutic effect in ulcerative colitis. Spexin suppresses oxidative damage and neutrophil infiltration in the acetic acid induced ulcerative colitis rat model. Spexin reduces inflammation in ulcerative colitis by inhibiting the NLRP3 inflammasome pathway. Spexin suppresses the NF‐κB‐mediated increase in pro‐inflammatory cytokines in colitis.

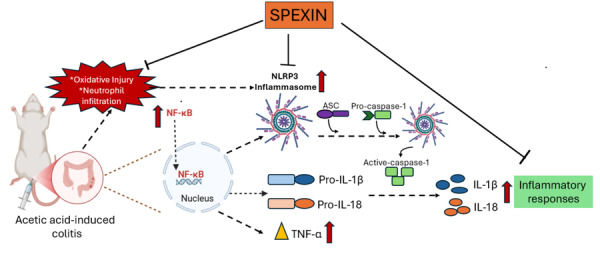

## 1 Introduction

Inflammatory bowel diseases (IBDs), including ulcerative colitis (UC), represent chronic gastrointestinal disorders characterized by persistent inflammation, diarrhea, abdominal discomfort, rectal bleeding, and unintended weight loss [1, 2]. The development of UC is multifaceted, involving an intricate interplay between genetic predisposition and environmental influences that disrupt the immune system, culminating in digestive and intestinal inflammation [2, 3].

The NOD‐like receptor pyrin domain‐containing 3 (NLRP3) inflammasome is a key element of the innate immune response and is critically implicated in the progression of inflammatory conditions such as colitis [4]. Its canonical activation follows two distinct signaling pathways [5]. The first, signal‐1, is initiated by pathogen‐associated molecular patterns (PAMPs) like lipopolysaccharides, which activate the nuclear factor‐κB (NF‐κB) pathway. This activation leads to the degradation of the inhibitor alpha (IκBα) in the NF‐κB complex, phosphorylation of the p65 subunit, and subsequent nuclear translocation [6]. NF‐κB p65 then promotes downstream NLRP3 activation and stimulates cytokine production, including pro‐ interleukin (IL)‐1β, pro‐IL‐18, and tumor necrosis factor (TNF)‐α, contributing to chronic inflammation and cell death [7]. In the second activation pathway of the NLRP3 inflammasome (signal‐2) is triggered by damage‐associated molecular patterns (DAMPs) such as adenosine triphosphate (ATP). This process activates potassium efflux, reactive oxygen species (ROS) formation, and lysosomal damage, ultimately inducing the assembly of the NLRP3 inflammasome [8, 9]. In contrast, the noncanonical NLRP3 pathway bypasses signal‐1, being directly activated by intracellular DAMPs like ROS. Both pathways drive the release of inflammatory cytokines, such as IL‐1β, IL‐18, and TNF‐α, which exacerbate mucosal inflammation in UC and elevate the risk of carcinogenesis [10]. Consequently, targeting NF‐κB or NLRP3 inflammasome activity, which regulates cytokines central to UC progression, is considered a promising therapeutic approach to mitigate disease severity and promote remission.

Spexin (SPX), also known as neuropeptide Q (NPQ), was recently identified using bioinformatics approaches aimed at discovering novel peptide composed of 14 amino acids [11, 12]. It is widely expressed in both central and peripheral tissues and binds to galanin receptor subtypes II and III (GALR2/3) [11, 13]. SPX is involved in regulating body weight, appetite, energy homeostasis, glucose and lipid metabolism, lipid storage, and arterial blood pressure [14]. Its levels are responsive to metabolic alterations and are frequently diminished in conditions like obesity, diabetes, and insulin resistance [15, 16, 17]. Therapeutically, SPX administration has demonstrated metabolic benefits, including reduced food intake, fat accumulation, and lipid levels, alongside anti‐inflammatory effects, improved insulin sensitivity, and enhanced energy expenditure [18, 19]. Although SPX has anti‐inflammatory effects through multiple pathways, there is limited information in the literature. The JAK2/STAT3 and GALR2 pathways regulate metabolic balance and lipid metabolism, reducing inflammation [20, 21]. The cAMP/PKA and MAPK pathways influence spexin expression via bile acid signaling, while the opioid receptor pathway mediates its antinociceptive effects [22, 23]. These mechanisms highlight spexin's potential as an anti‐inflammatory agent. However, there is a lack of research investigating the potential effects of spexin on ulcerative colitis and its mechanism. This study aims to explore the therapeutic impact and underlying mechanism of spexin on colonic injury induced by acetic acid in a model of ulcerative colitis.

## 2 The Methods & Materials

### 2.1 Animals

Sprague‐Dawley male rats (240–280 g, 10–12 weeks old) were supplied from the Sakarya University Animal Center. The rats were housed in a facility with a light/dark cycle (12/12 h) in which temperature (22 ± 2°C) and humidity (65%–70%) were controlled. They were provided standard pellet feed and water ad libitum, except for an 18‐h fasting period before the induction of colitis. The study protocol was confirmed by the Sakarya University Animal Care and Use Committee (approval number: 34, 06/07/2022), and all experimental procedures adhered to the guidelines of the New York Academy of Sciences and Turkish regulations on animal research.

### 2.2 Experimental Design and Colitis Induction

Rats were divided into experimental groups, with colitis induction achieved via intracolonic administration of acetic acid (AA). A polyethylene catheter (PE‐60), inserted 8 cm into the rectum under ether anesthesia, was used to deliver 1 mL of 5% AA solution. After 30 s, excess fluid was withdrawn, and the colon was flushed with saline [24]. Control animals received isotonic saline instead of AA. Post‐induction, colitis groups (*n* = 21) received intraperitoneal injections of saline, spexin (50 μg/kg/day, Phoenix Pharmaceuticals, 023‐81, USA), or sulfasalazine for 5 days. Control groups (*n* = 14) received either saline or spexin. On the sixth day, rats were anesthetized for intracardiac blood collection and euthanized, after which colonic tissues were harvested for analysis. The dose of spexin (50 µg/kg/day) was selected based on previous reports [25, 26].

### 2.3 Macroscopic Damage Scoring in the Colon

Excised colonic tissues were examined macroscopically, and damage was scored based on the mucosal condition: 0 = no damage; 1 = localized hyperemia without ulcers; 2 = single ulceration without inflammation; 3 = single ulceration with inflammation; 4 = ulceration areas < 1 cm with inflammation; 5 = ulceration areas ≥ 1 cm with inflammation [27].

### 2.4 Evaluation of Disease Activity Index

Disease activity was assessed following colitis induction by scoring stool consistency, the presence of blood in stool, and body weight loss. Scores were determined as follows: 0: weight loss none; 1%–5%: 1; 5%–10%: 2; 10%–20%: 3; > 20%: 4, stool consistency normal: 0; mildly soft: 1; very soft: 2; watery: 3, and blood presence negative: 0; fecal occult: 2; blood positive: 3; significant bleeding: 4 [28].

### 2.5 Assessment of Colonic Edema

An 8‐cm segment of the rectocolonic tissue was excised and opened longitudinally. The distal 6‐cm was weighed to calculate the colon weight/length ratio. The remaining 2‐cm proximal segment was used to determine the wet‐to‐dry weight ratio by drying the tissue at 80°C for 24 h and reweighing [27].

### 2.6 Measurement of Colonic Myeloperoxidase Activity, Malondialdehyde and Glutathione Levels

Myeloperoxidase (MPO) activity, indicating neutrophil infiltration, was measured spectrophotometrically at 460 nm using the H_2_O_2_‐dependent oxidation of o‐dianisidine. Results were expressed as units per gram of tissue (21). Lipid peroxidation was quantified via malondialdehyde (MDA) levels, measured at 535 nm by detecting thiobarbituric acid‐reactive substances, and expressed as nmol MDA per gram of tissue. Glutathione (GSH) content was determined via the modified Ellman method, measuring absorbance at 412 nm, with results presented as μmol GSH per gram of tissue [29].

### 2.7 Measurements of TNF‐α, IL‐1β and IL‐18 Levels in the Colon and Serum

Colonic levels of TNF‐α (E0764Ra, Bioassay Technology Laboratory), serum and colonic levels of IL‐1β (E0119Ra, Bioassay Technology Laboratory) and IL‐18 (E0117Ra, Bioassay Technology Laboratory) were determined by using the rat ELISA kits according to the manufacturer's procedure. Protein concentrations in the colonic tissue were determined by the BCA kit (E‐BC‐K318‐M, Elabscience).

### 2.8 Western Blot Analyses for Protein Expressions of NF‐κB p65, NLRP3 and Caspase‐1 in the Colon

Proteins (25 μg per sample) were separated by 12% SDS‐PAGE, transferred to nitrocellulose membranes, and blocked with nonfat milk in Tris‐buffered saline (TBS) [30]. Membranes were incubated overnight at 4°C with primary antibodies targeting NF‐κB p65 (E‐AB‐22066, Elabscience, 1:500), NLRP3 (PA5‐79740, Invitrogen, 1:500), cleaved caspase‐1 (STJ90021, St John's Laboratory, 1:500), and GAPDH (loading control, Santa Cruz, 1:1000). Following TBS‐T washes, membranes were incubated with HRP‐conjugated secondary antibodies (Bio‐Rad, USA) for 1 h. Protein bands were visualized using enhanced chemiluminescence (Bio‐Rad, USA).

### 2.9 Microscopic Examination of the Colon Samples

Colonic tissues were fixed in 10% formaldehyde, dehydrated in graded alcohols, cleared with toluene, and embedded in paraffin. Sections (5 μm) were stained with hematoxylin and eosin and evaluated under an Olympus BX51 microscope. Histological scoring was performed based on four parameters: damage/necrosis (0 = absent; 1 = localized; 2 = moderate; 3 = severe), submucosal edema (0 = absent; 1 = mild; 2 = moderate; 3 = severe), inflammatory cell infiltration (0 = absent; 1 = mild; 2 = moderate; 3 = severe), and vasculitis (0 = absent; 1 = mild; 2 = moderate; 3 = severe). Scores were combined for a maximum total of 13 [31].

### 2.10 Statistical Analysis

Group differences were assessed using one‐way ANOVA followed by Bonferroni post‐hoc tests in GraphPad Prism 9.3.0. Data were presented as mean ± standard error of the mean (SEM), with statistical significance defined as *p* < 0.05.

## 3 Results

**FIGURE 1:**
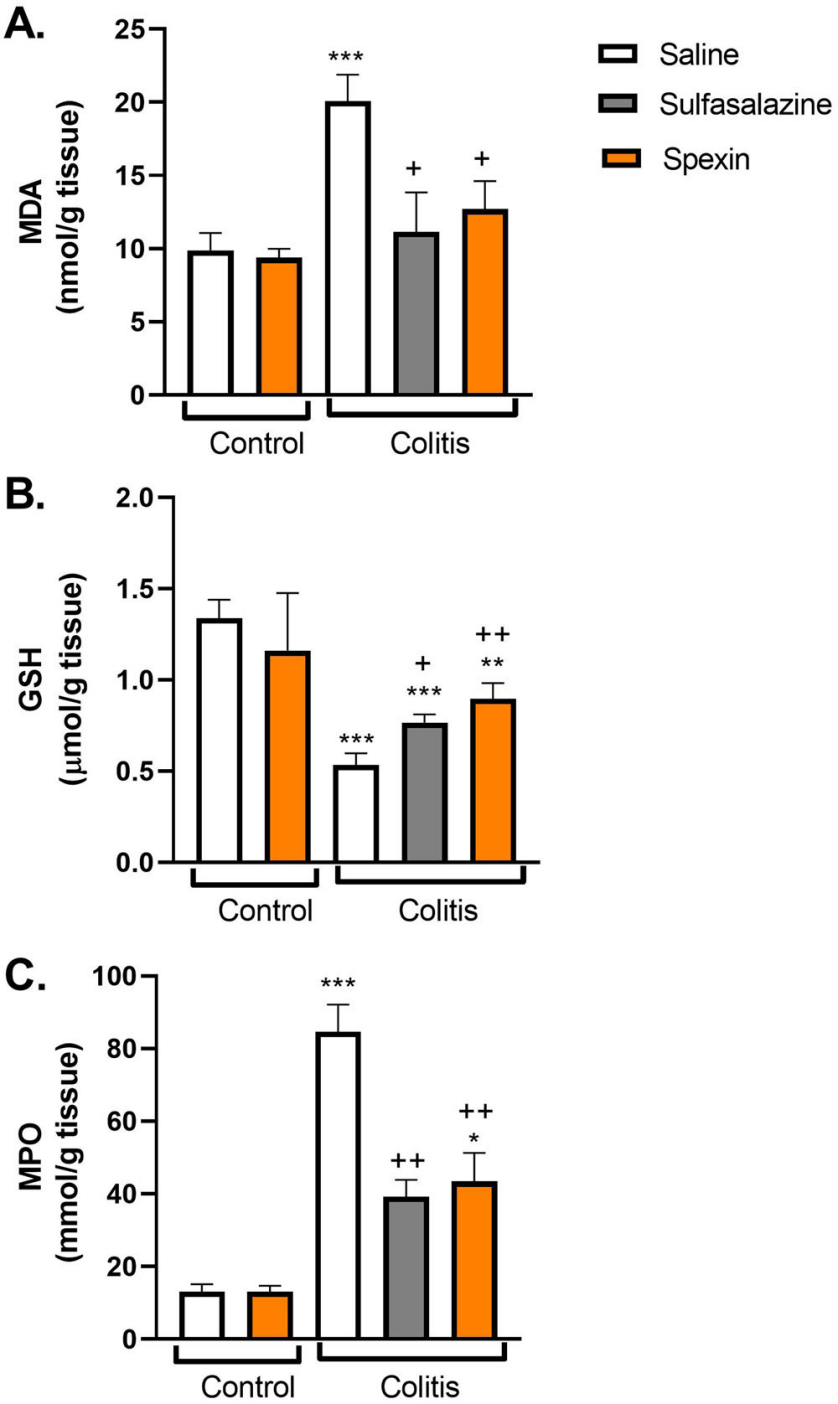
(A) Malondialdehyde (MDA), (B) Glutathione (GSH) and (C) Myeloperoxidase (MPO) activity levels in colon tissues of experimental groups. Results represent the mean ± S.E.M. Each group consists of seven rats. **p < 0.01, ***p < 0.001, * compared to saline‐treated control group; ^+^ *p* < 0.05, ^++^ *p* < 0.01, ^+^ compared to saline‐treated colitis group.

The macroscopic damage score was significantly increased in the colitis‐induced group treated with saline compared to the saline‐treated control group (*p* < 0.001, Table 1). This increase was attenuated in the treatment group receiving sulfasalazine (*p* < 0.05). Similarly, spexin treatment effectively suppressed colitis‐induced damage, resulting in a significant reduction in the macroscopic damage score (*p* < 0.01) and ulcerative areas in colon (Supporting Information S1: Figure 1). When edema was evaluated in the colon tissue, an increase in the wet‐to‐dry weight difference was observed with the development of colitis (*p* < 0.01). Compared to saline‐treated colitis group, spexin treatment effectively suppressed the edema associated with colitis (*p* < 0.01). Also, this increase was reduced in the sulfasalazine treatment (*p* < 0.001). In the control group treated with spexin, no damage was observed in colonic tissue. When the weight change of the rats was evaluated at the end of the experiment, no significant difference was determined. It was observed that the stool consistency increased with the formation of colitis compared to the saline‐treated control and this increase was reduced by both sulfasalazine and spexin treatment (*p* < 0.01–0.001). No blood was found in the stool in all experimental groups.

**TABLE 1:** Effect of spexin on macroscopic damage score, colonic edema and disease activity index (DAI) parameters in acetic acid‐induced colitis and control groups.

Score parameters	Control		Colitis		
	Saline	Spexin (50 µg.kg^−1^)	Saline	Sulfasalazine	Spexin (50 µg.kg^−1^)
Macroscopic score	0 ± 0	0 ± 0	**2.66** ± **0.33** ^***^	**1.40** ± 0.24* ^**+**^	**1.20** ± **0.20** ^**++**^
Colonic wet–dry weight ratio	0.13 ± 0.004	0.15 ± 0.01	**0.19** ± **0.01** ^**^	**0.12** ± **0.006** ^**+++**^	**0.14** ± **0.01** ^**++**^
Weight change (gr)	24.83 ± 2.21	27.50 ± 2.47	20.00 ± 1.50	20.00 ± 1.76	18.60 ± 4.79
Stool consistency	0 ± 0	0 ± 0	2.66 ± 0.21	**1.00** ± **0.31** ^**+++**^	**1.40** ± **0.24** ^**++**^
Blood in stool	None	None	None	None	None

^+^ *p* < 0.05, ^++^ *p* < 0.01, ^+++^ *p* < 0.001; ^+^vs saline‐treated colitis group.

Malondialdehyde (MDA) and myeloperoxidase (MPO) levels were significantly elevated in the colitis‐induced group compared to controls (*p* < 0.001), while glutathione (GSH) levels were markedly decreased (*p* < 0.001, Figure 1). Spexin treatment significantly reduced MDA and MPO levels (*p* < 0.05 and *p* < 0.01, respectively) and restored GSH content (*p* < 0.01). Sulfasalazine treatment produced similar effects, though GSH restoration was less pronounced compared to spexin.

**FIGURE 2:**
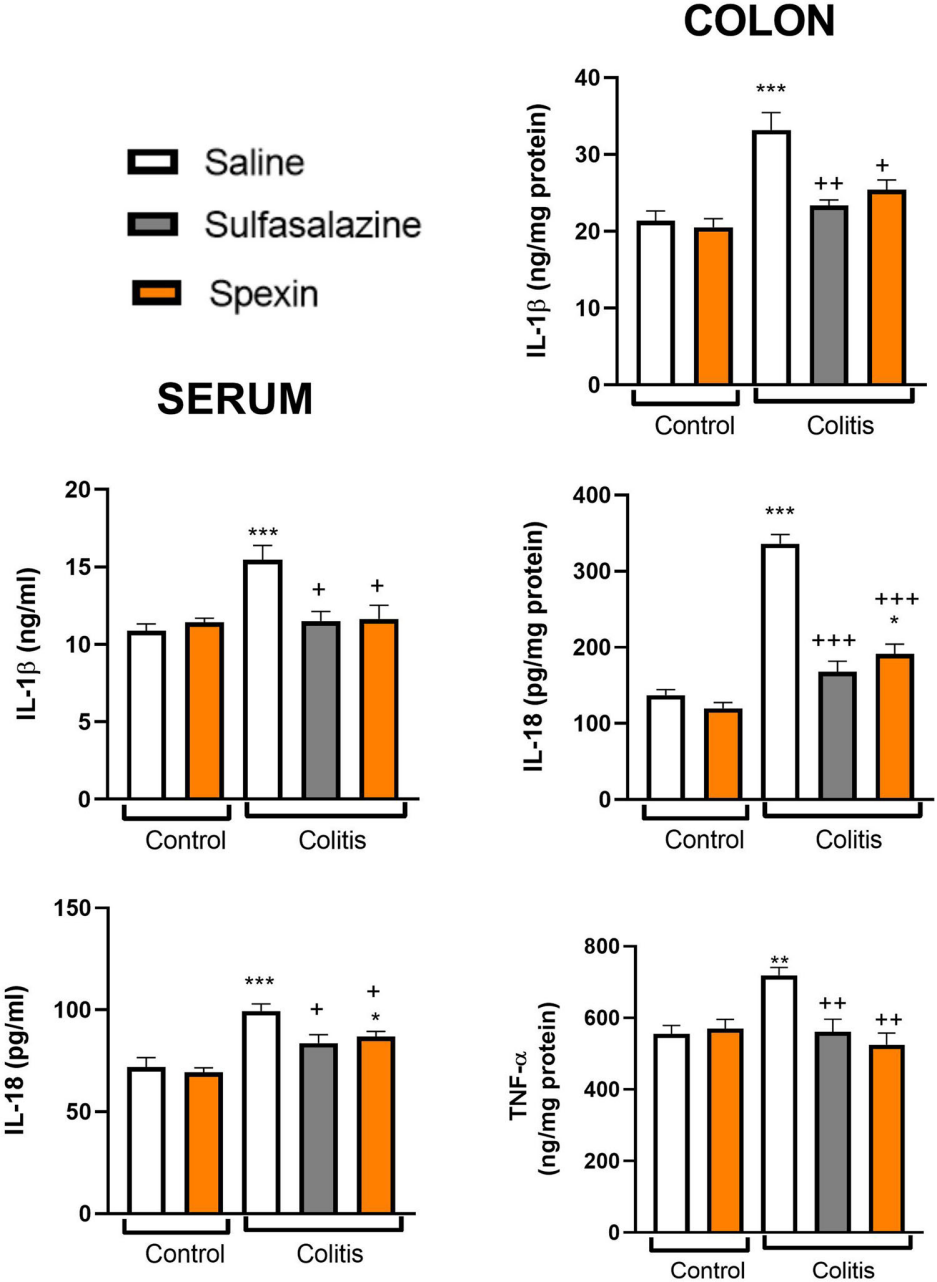
The colonic TNF‐α levels, and IL‐18 and IL‐1β levels in serum and colon samples of experimental groups. Results represent the mean ± S.E.M. Each group consists of seven rats. *p < 0.05, **p < 0.01, ***p < 0.001, * compared to saline‐treated control group; ^+^ *p* < 0.05, ^++^ *p* < 0.01, ^+++^ *p* < 0.001, ^+^ compared to saline‐treated colitis group.

The levels of pro‐inflammatory cytokines TNF‐α, IL‐1β and IL‐18 were not significantly affected by spexin administration in the control group. However, the induction of colitis led to a significant increase in both serum and colonic IL‐1β and IL‐18 levels compared to the control group (*p* < 0.001, Figure 2). Treatment with sulfasalazine suppressed cytokine levels in both serum and colonic tissues compared to the colitis group (*p* < 0.05–0.001). Similarly, spexin administration resulted in a significant reduction in the pro‐inflammatory cytokines including colonic TNF‐α, IL‐1β and IL‐18 levels in both serum and colonic tissues (*p* < 0.05–0.001). These findings suggest that spexin effectively alleviated colonic inflammation.

**FIGURE 3:**
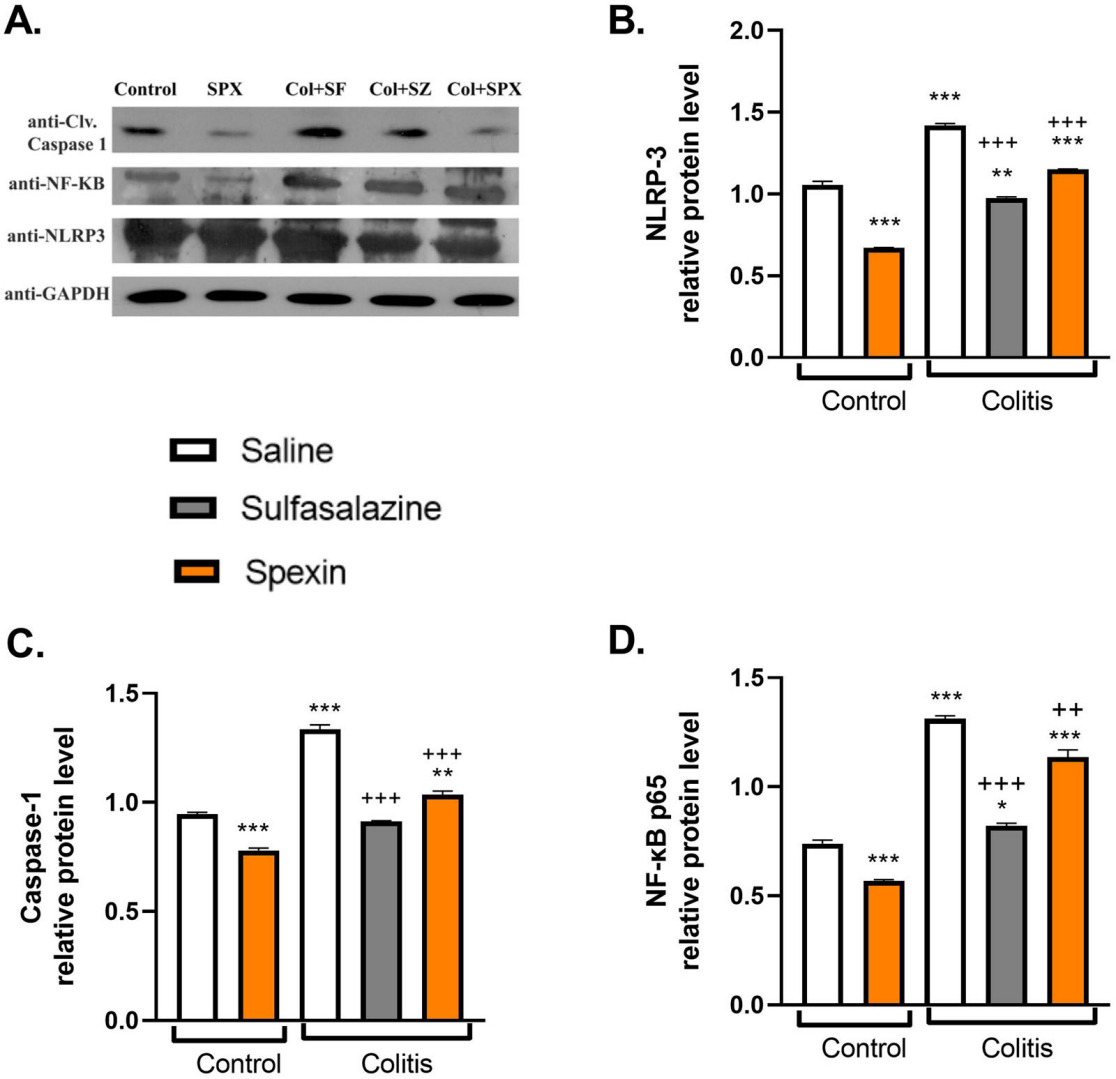
Effect of Spexin on NLRP3, caspase‐1 and NF‐κB p65 protein levels in acetic acid‐induced ulcerative colitis. (A) Representative Western blot bands of protein levels. (B–D) NLRP3, caspase‐1 and NF‐κB protein expression levels in the colon samples, respectively. *p < 0.05, **p < 0.01, ***p < 0.001, * compared to saline‐treated control group; ^++^ *p* < 0.01, ^+++^ *p* < 0.001, ^+^ compared to saline‐treated colitis group.

When spexin was administered to the control group, a decrease in NLRP3, NF‐κB, and caspase‐1 protein levels was observed compared to the control group receiving physiological saline (*p* < 0.001, Figure 3). Compared to the control group, Colitis induction resulted in significant upregulation of NF‐κB p65, NLRP3, and caspase‐1 protein levels (*p* < 0.001). Spexin treatment significantly suppressed these proteins (*p* < 0.01–0.001), indicating inhibition of the NF‐κB/NLRP3 inflammasome pathway. Sulfasalazine also reduced these protein levels (*p* < 0.001).

**FIGURE 4:**
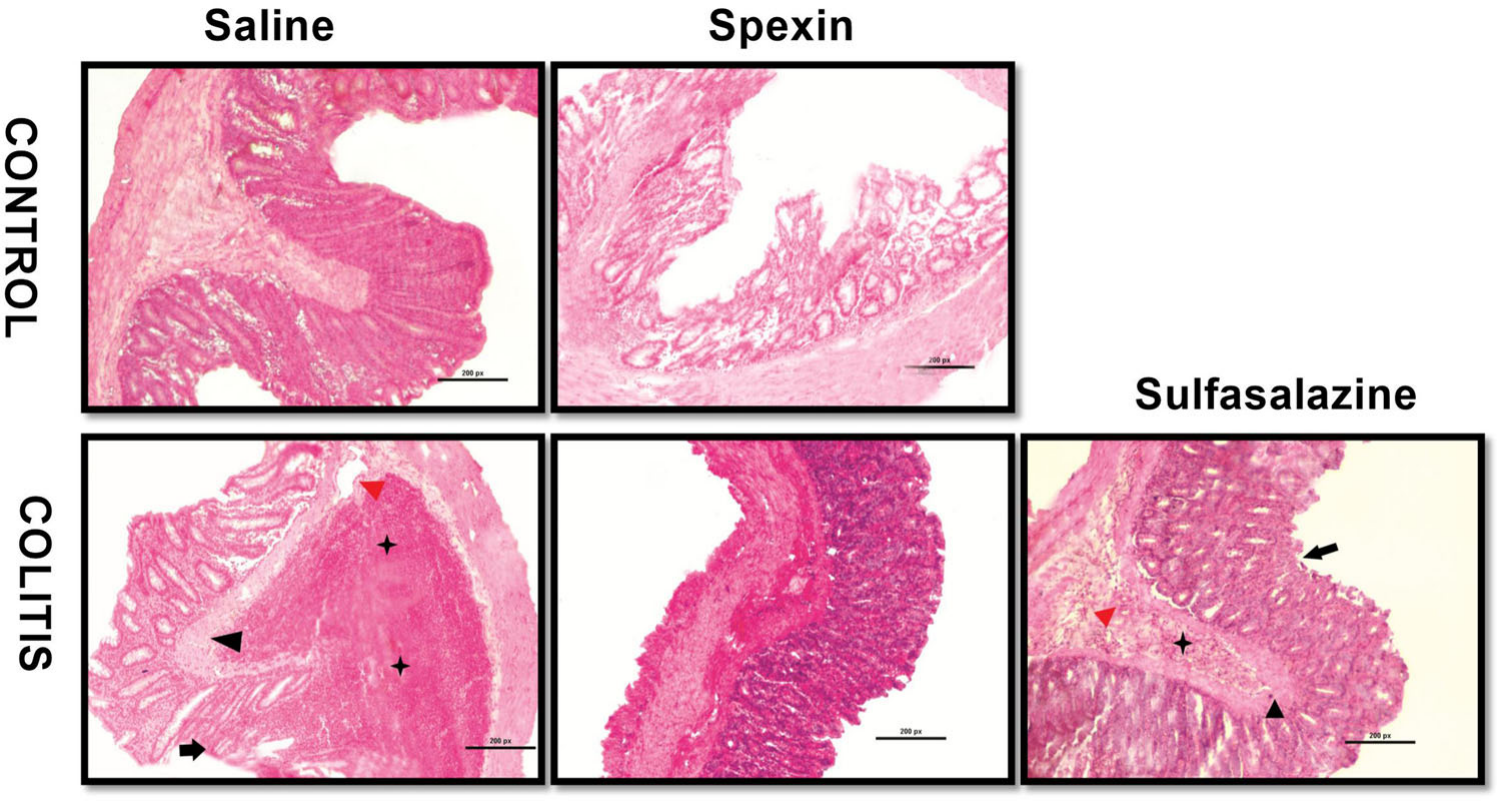
Representative colon microscopic images of experimental groups. Black arrow: Damage/necrosis; black arrowhead: Submucosal edema; black star: inflammatory cell infiltration; red arrowhead: vasculitis, Hematoxylin and eosin, Magnification: 20×; Scale bar: 200 μm.

**FIGURE 5:**
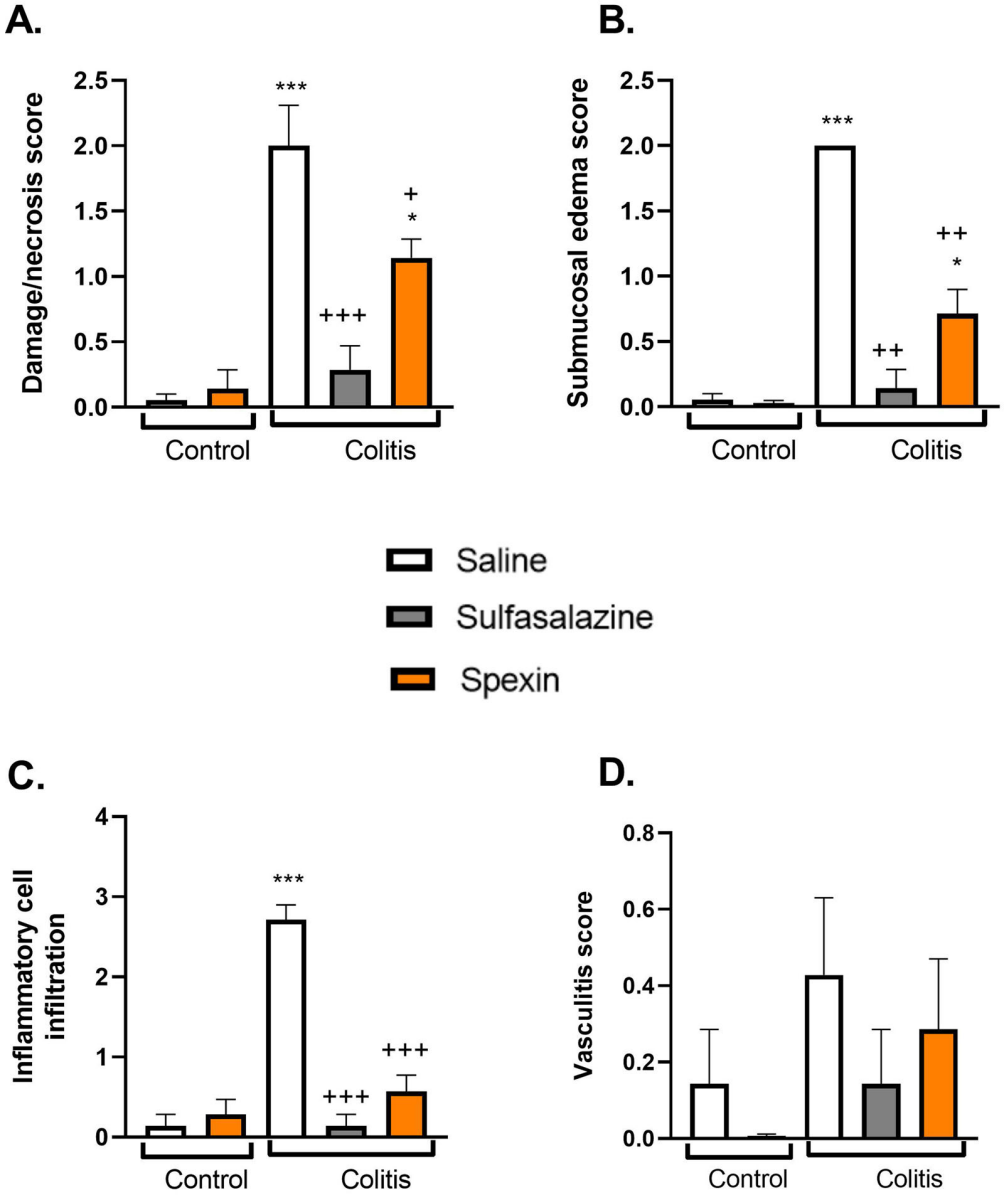
Histopathological scoring results of experimental groups. (A) Damage/necrosis score, (B) Submucosal edema score, (C) Inflammatory cell infiltration, (D) Vasculitis score. Results represent the mean ± S.E.M. Each group consists of seven rats. *p < 0.05, ***p < 0.001, * compared to saline‐treated control group; ^+^ *p* < 0.05, ^++^ *p* < 0.01, ^+++^ *p* < 0.001, ^+^ compared to saline‐treated colitis group.

According to the histological evaluation of the groups, no findings of mucosal damage/necrosis, edema, submucosal thickening, or hemorrhage were observed in the colon samples obtained from the control groups treated with saline or spexin. The scores for these groups were determined to be at most one (Figures 4 and 5). In contrast, the colitis group exhibited the highest scores, with severe deterioration observed in all parameters. Notably, significant increases were detected in inflammatory cell infiltration, submucosal edema, and damage/necrosis scores in the colitis group (*p* < 0.001). In the spexin‐treated colitis group, significant reductions in inflammation, edema, submucosal thickening, and hemorrhage scores were observed (*p* < 0.05–0.001). Although the histological structure of the spexin‐treated colitis group did not completely match that of the control group, noticeable improvements and areas resembling normal histological structure were observed. In the sulfasalazine group, designed as a positive control, low damage scores were recorded in all evaluations (*p* < 0.01–0.001).

## 4 Discussion

The findings of the current study reveal that rectal administration of acetic acid to induce colitis leads to colonic injury, characterized by elevated levels of pro‐inflammatory cytokines, increased neutrophil infiltration, apoptosis, oxidative damage, and activation of NLRP3 inflammasome signaling, accompanied by a depletion of the antioxidant GSH in colonic tissue. Spexin treatment effectively suppressed colonic oxidative injury, reduced neutrophil infiltration, and attenuated pro‐inflammatory cytokine responses. Additionally, spexin decreased colonic edema and microscopic damage scores while preserving colonic GSH levels. Furthermore, spexin treatment significantly reduced elevated protein levels of NLRP3, NF‐κB, and caspase‐1. These findings for the first time show that spexin exerts therapeutic effects against acetic acid‐induced oxidative and inflammatory colonic injury in rats by modulating the NLRP3 inflammasome pathway.

Ulcerative colitis (UC) is a chronic inflammatory disease of the colonic mucosa, commonly presenting with symptoms such as bloody diarrhea, rectal urgency, and mucosal inflammation of the colon [32]. While acetic acid‐induced inflammation in rodents does not perfectly replicate human ulcerative colitis, it shares common pathological characteristics such as colonic inflammation, epithelial erosions, enhanced vascular permeability, and neutrophil infiltration [27, 33]. In colitis induced by acetic acid, elevated oxidative stress, reflected by increased lipid peroxidation and decreased antioxidant defenses, is accompanied by the presence of inflammation and the activation of pro‐inflammatory mediators [34]. Inflammation and oxidative damage are thought to play the most important role in the pathophysiological process of ulcerative colitis [35]. The formation of oxidative stress and low antioxidant capacities are associated with the pathogenesis of ulcerative colitis, and these biochemical changes may increase the symptoms and complications of the disease due to the harmful effects of oxidants and free radicals on cellular structures [36]. Consistent with prior studies, our findings confirm that acetic acid‐induced colitis leads to oxidative stress, evidenced by elevated lipid peroxidation and reduced antioxidant capacity in colonic tissues. Similarly, the development of colitis in rats caused significant decreases in GSH, superoxide dismutase and catalase activities, confirming that the antioxidant capacity of colonic tissue is depleted during colonic inflammation [27, 37]. On the other hand, spexin treatment suppresses MDA levels, a marker of oxidative damage associated with colitis, and leads to an increase in GSH content, highlighting its therapeutic effects against oxidative stress. Previous studies reported that spexin treatment reduced oxidative stress markers in kidney tissue in a high fat/fructose diet‐induced obesity model [38] and attenuated doxorubicin‐induced myocardial MDA elevation [39].

Myeloperoxidase (MPO) activity from fecal and serum samples has been demonstrated to elevate in patients with ulcerative colitis, reflecting the inflammatory state of the disease [40, 41]. Elevated MPO levels are commonly associated with increased neutrophil infiltration [42]. The measurement of MPO activity serves as a useful biomarker for assessing the degree of inflammation and oxidative stress in these patients, as its activity is directly linked to the intensity of the inflammatory response. Therefore, compounds that can prevent the recruitment of neutrophils to the inflamed colon may offer potential therapeutic benefits in managing inflammatory damage [43]. The current study revealed that spexin treatment resulted in a reduction of MPO activity, which is elevated in colitis, suggesting an inhibitory effect of spexin on neutrophil accumulation during the chronic oxidative process of colitis. The potential effect of spexin on MPO activity was demonstrated for the first time in our study. Spexin is a peptide involved in various physiological processes, including regulating inflammation and immune responses [14, 18]. Some studies suggest that spexin has anti‐inflammatory effects, potentially through its influence on immune cell signaling and modulation of oxidative stress [38, 44]. It is possible that spexin could influence MPO activity indirectly by reducing the infiltration of neutrophils or modulating the inflammatory response. However, more direct studies are needed to confirm whether spexin specifically affects MPO activity or its role in inflammatory conditions.

Nod‐like receptor pyrin domain‐1 containing 3 (NLRP3) inflammasome/nuclear factor kappa B (NF‐κB) inflammation signaling is known to be involved in the pathogenesis of UC [45]. The NF‐κB heterodimer complex resides in the cytosol, where it triggers the degradation of the NFκB inhibitor alpha (IκBα) subunit, followed by the phosphorylation of the NF‐κB p65 subunit and its subsequent translocation into the nucleus here, it activates the transcription of downstream target genes such as NLRP3 inflammasome proteins, and causes chronic inflammation and cell apoptosis [6, 46].

NF‐κB is a ubiquitously expressed transcription factor that governs the expression of genes linked to immune modulation, cell movement, inflammation, and apoptosis. The activation of NF‐κB plays a pivotal role in driving inflammation by upregulating pro‐inflammatory cytokines such as TNF‐α, contributing to disease progression [47]. In patients with ulcerative colitis, TNF‐α levels are significantly elevated, contributing to chronic inflammation and mucosal injury [48, 49]. Similarly, in the acetic acid‐induced ulcer model, both NF‐κB activation and TNF‐α expression are markedly increased, contributing the inflammatory and oxidative responses observed in ulcerative colitis [50]. Given the complex pathogenesis of UC, current therapeutic approaches often result in either inadequate treatment response or significant adverse effects in UC patients [51]. This underscores the necessity for the development of more effective treatment strategies, with a primary focus on the suppression of inflammation [52, 53]. Our results demonstrated that treatment of UC rats with spexin significantly reduced the increased colonic concentrations of TNF‐α, NF‐κB, IL‐1β and IL‐18. Given that IL‐1β and IL‐18 are downstream products of NLRP3 inflammasome activation, the reduction of these cytokines in our study suggests that spexin may exert its effects by modulating NLRP3 inflammasome activity. Clinical studies have shown that spexin levels are significantly lower in obese individuals [54]. Obesity is considered a chronic state of systemic inflammation, and the reduced levels of spexin may indicate its potential impact on the regulation of inflammation. Preclinical studies reported that spexin suppresses lipid peroxidation, enhances antioxidant capacity, and exerts an anti‐inflammatory effect by reducing serum IL‐1β and TNF‐α levels in rats with type 2 diabetes [55]. It also has been found to suppress serum TNF‐α and IL‐6 levels in a rat model of metabolic syndrome induced by a high‐fructose diet [18]. Gambora et al. (2020) showed that spexin ameliorated adipose tissue inflammation and macrophage recruitment by suppressing epididymal TNF‐α, IL1‐β and IL‐6 expression and macrophages in obese mice [44]. Central application of spexin caused an antinociceptive effect for acute inflammatory pain [23].

Activation of the NLRP3 inflammasome leads to the recruitment of the adaptor protein apoptosis‐associated speck‐like protein containing a C‐terminal caspase recruitment domain (ASC), which subsequently facilitates the cleavage and activation of pro‐caspase‐1 into its active form [56]. The activated caspase‐1 then processes the inactive precursors pro‐IL‐1β and pro‐IL‐18 into their active forms, the pro‐inflammatory cytokines IL‐1β and IL‐18 [57]. These cytokines are key mediators of intestinal inflammation, promoting immune cell infiltration, epithelial barrier disruption, and tissue damage observed in colitis [58]. Dysregulation of the NLRP3 inflammasome has been implicated in the exacerbation of colonic inflammation, making it a potential therapeutic target for controlling inflammation and oxidative damage in ulcerative colitis [59].

Overactivity of the NLRP3 inflammasome has been reported to increase inflammation, and damage to the intestinal mucosa, disrupting the intestinal mucosal barrier [38]. Studies have shown that the NLRP3 inflammasome triggers inflammatory processes in the colon and that dysregulation of this inflammasome may lead to various pathologies such as ulcerative colitis and colitis‐associated colon cancer [60, 61]. In contrast to these studies, Itani et al. (2016) demonstrated that NLRP3 inflammasome activation plays a protective role by reducing intestinal inflammation and damage in an oxazolone‐induced colitis model [62]. Contrary to these findings, suppression of NLRP3 inflammasome activation against acetic acid‐induced ulcerative colitis has been shown to attenuate inflammation by reducing the levels of inflammatory markers and oxidative stress [35]. Similarly, our results demonstrate for the first time that spexin treatment suppresses the NF‐κB/NLRP3 inflammasome signaling pathway and reduces pro‐inflammatory cytokine levels (TNF‐α, IL‐beta and IL‐18). It has been determined that spexin reduces the gene expression levels of IL‐1β, IL‐17A, IL‐18, IL‐33, ALOX15, COX‐1, COX‐2, TGF‐β1, TNF‐α in kidney tissue in chronic kidney damage [63]. Spexin showed protective effects on systemic inflammation and kidney damage by suppressing inflammatory cytokine levels in adenine‐induced chronic renal failure rat model [64]. However, its relationship with the NLRP3 inflammasome pathway has not been found in the literature.

## 5 Conclusion

Our study demonstrates that exogenous spexin plays an important role in suppressing cellular oxidant damage and inflammation in the ulcerative colitis model. In this process, spexin exhibited healing effects by suppressing the activation of the NLRP3 inflammasome and the release of pro‐inflammatory cytokines associated with this signaling pathway. Additionally, the mechanism of action of spexin and its relationship with the NF‐κB/NLRP3 signaling pathway were revealed for the first time. We believe that spexin may be a potential target for developing new treatment strategies in ulcerative colitis and that further studies are needed.

While our study provides valuable insights into the therapeutic potential of spexin in ulcerative colitis, certain limitations should be considered. The acetic acid‐induced colitis model in rats, while commonly used and similar to human ulcerative colitis in some ways, may not fully capture the complexity of the disease. Its effects on different experimental colitis models can be tested. Additionally, our study primarily focused on short‐term effects, and further investigations are required to evaluate the long‐term effects of spexin treatment.

## Author Contributions

All the experiments were performed at the Sakarya University School of Medicine, Departments of Physiology and Histology & Embryology, Sakarya, Türkiye. All individuals who meet the criteria for authorship are listed. All authors have agreed to be accountable for all aspects of the work in ensuring that questions related to the accuracy or integrity of any part of the work are appropriately investigated and resolved. Design of work: Sevil Arabacı Tamer. Performing the experiments and data acquisition: Sevil Arabacı Tamer, Fadime Köse. Data interpretation and application of statistical analysis: Sevil Arabacı Tamer, Fadime Köse, Sevinç Yanar, Özcan Budak, Cahit Bağcı (all authors). Drafting of the manuscript: Sevil Arabacı Tamer, Fadime Köse, Sevinç Yanar, Özcan Budak, Cahit Bağcı (all authors). Critical revision of the manuscript: Sevil Arabacı Tamer. Approval of the final version of the manuscript: Sevil Arabacı Tamer, Fadime Köse, Sevinç Yanar, Özcan Budak, Cahit Bağcı (all authors).

## Ethics Statement

The present study was approved by the Ethics Committee of Sakarya University.

## Conflicts of Interest

The authors declare no conflicts of interest.

## Supporting information

suplm. Fig. 1.pdf.

## Data Availability

The authors have nothing to report.

## References

[1] G. Amodeo , S. Franchi , G. Galimberti , B. Riboldi , and P. Sacerdote , “The Prokineticin System in Inflammatory Bowel Diseases: A Clinical and Preclinical Overview,” Biomedicines 11, no. 11 (2023): 2985. 38001985 10.3390/biomedicines11112985 PMC10669895

[2] H. Arya , R. Dass , B. Chopra , et al., “An Update on Herbal Products for the Management of Inflammatory Bowel Disease,” Anti‐Inflammatory & Anti‐Allergy Agents in Medicinal Chemistry 22, no. 1 (2023): 1–9. 37497699 10.2174/1871523022666230727094250

[3] Z. Che , Z. Ye , X. Zhang , et al., “Mesenchymal Stem/Stromal Cells in the Pathogenesis and Regenerative Therapy of Inflammatory Bowel Diseases,” Frontiers in Immunology 13 (2022): 952071, 10.3389/fimmu.2022.952071. 35990688 PMC9386516

[4] N. Dai , X. Yang , P. Pan , et al., “Bacillus Paralicheniformis, an Acetate‐Producing Probiotic, Alleviates Ulcerative Colitis via Protecting the Intestinal Barrier and Regulating the NLRP3 Inflammasome,” Microbiological Research 287 (2024): 127856. 39079268 10.1016/j.micres.2024.127856

[5] A. P. Perera , K. Sajnani , J. Dickinson , R. Eri , and H. Körner , “NLRP3 Inflammasome in Colitis and Colitis‐Associated Colorectal Cancer,” Mammalian Genome 29 (2018): 817–830. 30206651 10.1007/s00335-018-9783-2

[6] Q. Guo , Y. Jin , X. Chen , et al., “NF‐κB in Biology and Targeted Therapy: New Insights and Translational Implications,” Signal Transduction and Targeted Therapy 9, no. 1 (2024): 53. 38433280 10.1038/s41392-024-01757-9 PMC10910037

[7] T. Liu , L. Zhang , D. Joo , and S. C. Sun , “NF‐κB Signaling in Inflammation,” Signal Transduction and Targeted Therapy 2, no. 1 (2017): 17023. 29158945 10.1038/sigtrans.2017.23 PMC5661633

[8] C. Jin and R. A. Flavell , “Molecular Mechanism of NLRP3 Inflammasome Activation,” Journal of Clinical Immunology 30 (2010): 628–631. 20589420 10.1007/s10875-010-9440-3

[9] S. Wang , Y. Lin , X. Yuan , F. Li , L. Guo , and B. Wu , “REV‐ERBα Integrates Colon Clock With Experimental Colitis Through Regulation of NF‐κB/NLRP3 Axis,” Nature Communications 9, no. 1 (2018): 4246. 10.1038/s41467-018-06568-5 PMC6185905 30315268

[10] Y. Zhen and H. Zhang , “NLRP3 Inflammasome and Inflammatory Bowel Disease,” Frontiers in Immunology 10 (2019): 276. 30873162 10.3389/fimmu.2019.00276 PMC6403142

[11] O. Mirabeau , E. Perlas , C. Severini , et al., “Identification of Novel Peptide Hormones in the Human Proteome by Hidden Markov Model Screening,” Genome Research 17, no. 3 (2007): 320–327. 17284679 10.1101/gr.5755407 PMC1800923

[12] K. Sonmez , N. T. Zaveri , I. A. Kerman , et al., “Evolutionary Sequence Modeling for Discovery of Peptide Hormones,” PLoS Computational Biology 5, no. 1 (2009): e1000258. 19132080 10.1371/journal.pcbi.1000258 PMC2603333

[13] D.‐K. Kim , S. Yun , G. H. Son , et al., “Coevolution of the Spexin/Galanin/Kisspeptin Family: Spexin Activates Galanin Receptor Type II and III,” Endocrinology 155, no. 5 (2014): 1864–1873. 24517231 10.1210/en.2013-2106

[14] İ. Türkel , G. Memi , and B. Yazgan , “Impact of Spexin on Metabolic Diseases and Inflammation: An Updated Minireview,” Experimental Biology and Medicine 247, no. 7 (2022): 567–573. 35068225 10.1177/15353702211072443 PMC9014522

[15] T. Chen , F. Wang , Z. Chu , et al., “Circulating Spexin Decreased and Negatively Correlated With Systemic Insulin Sensitivity and Pancreatic β Cell Function in Obese Children,” Annals of Nutrition and Metabolism 74, no. 2 (2019): 125–131. 30673665 10.1159/000496459

[16] S. E. Gambaro , M. G. Zubiría , A. P. Giordano , et al., “Role of Spexin in White Adipose Tissue Thermogenesis Under Basal and Cold‐Stimulated Conditions,” International Journal of Molecular Sciences 25, no. 3 (2024): 1767. 38339044 10.3390/ijms25031767 PMC10855774

[17] Y. Liu , D. Wu , H. Zheng , et al., “Serum Spexin Level Is Negatively Associated With Peripheral Neuropathy and Sensory Pain in Type 2 Diabetes,” Journal of Diabetes Research 2024 (2024): 4538199. 38919263 10.1155/2024/4538199 PMC11199070

[18] M. A. Said , N. Y. Nafeh , and H. A. Abdallah , “Spexin Alleviates Hypertension, Hyperuricaemia, Dyslipidemia and Insulin Resistance in High Fructose Diet Induced Metabolic Syndrome in Rats via Enhancing PPAR‐ɣ and AMPK and Inhibiting IL‐6 and TNF‐α,” Archives of Physiology and Biochemistry 129, no. 5 (2023): 1111–1116. 33721543 10.1080/13813455.2021.1899242

[19] M. Yu , M. Wang , S. Han , et al., “Spexin Ameliorates Skeletal Muscle Insulin Resistance Through Activation of GAL2 Receptor,” European Journal of Pharmacology 917 (2022): 174731. 34973950 10.1016/j.ejphar.2021.174731

[20] M. Wang , Z. Zhu , Y. Kan , et al., “Treatment With Spexin Mitigates Diet‐Induced Hepatic Steatosis In Vivo and In Vitro Through Activation of Galanin Receptor 2,” Molecular and Cellular Endocrinology 552 (2022): 111688. 35654225 10.1016/j.mce.2022.111688

[21] B. Zeng , Q. Shen , B. Wang , et al., “Spexin Ameliorated Obesity‐Related Metabolic Disorders Through Promoting White Adipose Browning Mediated by JAK2‐STAT3 Pathway,” Nutrition & Metabolism 21, no. 1 (2024): 22. 38658956 10.1186/s12986-024-00790-3 PMC11040786

[22] Q. Lai , Y. Ma , J. Bai , et al., “Mechanisms for Bile Acids CDCA‐ and DCA‐Stimulated Hepatic Spexin Expression,” Cells 11, no. 14 (2022): 2159. 35883602 10.3390/cells11142159 PMC9316865

[23] S. Y. Lv , B. Cui , Y. Yang , et al., “Spexin/NPQ Induces FBJ Osteosarcoma Oncogene (Fos) and Produces Antinociceptive Effect Against Inflammatory Pain in the Mouse Model,” American Journal of Pathology 189, no. 4 (2019): 886–899. 30664863 10.1016/j.ajpath.2018.12.009

[24] Z. N. Özdemir , G. Tazegül , P. Kuru , et al., “Nicotine Alleviates Colitis‐Induced Damage in Rats via Its Anti‐Oxidative Activity,” Marmara Medical Journal 27, no. 1 (2014): 13–20.

[25] A. E. Atici , S. Arabacı Tamer , H. N. Levent , et al., “Neuropeptide W Attenuates Oxidative Multi‐Organ Injury in Rats Induced With Intra‐Abdominal Sepsis,” Inflammation 45, no. 1 (2022): 279–296. 34564825 10.1007/s10753-021-01545-5

[26] S. A. Tamer , S. Akbulut , O. Erdoğan , et al., “Protective Effects of Neuropeptide‐W on Stress‐Induced Gastric Ulcer in Rats,” in *Acta Physiologica*, (Wiley, 2019).

[27] S. Arabacı Tamer , S. Akbulut , Ö. Erdoğan , Ö. Çevik , F. Ercan , and B. Ç. Yeğen , “Neuropeptide W Exhibits Preventive and Therapeutic Effects on Acetic Acid‐Induced Colitis via Modulation of the Cyclooxygenase Enzyme System,” Digestive Diseases and Sciences 68, no. 6 (2023): 2441–2453. 36631709 10.1007/s10620-022-07811-2

[28] H. S. Cooper , S. N. Murthy , R. S. Shah , and D. J. Sedergran , “Clinicopathologic Study of Dextran Sulfate Sodium Experimental Murine Colitis,” Laboratory Investigation; A Journal of Technical Methods and Pathology 69, no. 2 (1993): 238–249. 8350599

[29] S. Arabacı Tamer , S. Akbulut , İ. Peker Eyüboğlu , et al., “Peripheral Administration of Neuropeptide‐W Protects Against Stress‐Induced Gastric Injury in Rats,” Life Sciences 310 (2022): 121087. 36252697 10.1016/j.lfs.2022.121087

[30] M. G. B. Albayrak , T. Simsek , M. Kasap , G. Akpinar , N. Z. Canturk , and S. A. Guler , “Tissue Proteome Analysis Revealed an Association Between Cancer, Immune System Response, and the Idiopathic Granulomatous Mastitis,” Medical Oncology 39, no. 12 (2022): 238. 36175807 10.1007/s12032-022-01845-2

[31] M. Gue , C. Bonbonne , J. Fioramonti , et al., “Stress‐Induced Enhancement of Colitis in Rats: CRF and Arginine Vasopressin Are Not Involved,” American Journal of Physiology‐Gastrointestinal and Liver Physiology 272, no. 1 (1997): G84–G91. 10.1152/ajpgi.1997.272.1.G84 9038880

[32] A. W. Uździcki and M. Wawrzynowicz‐Syczewska , “Characteristic Features of Ulcerative Colitis With Concomitant Primary Sclerosing Cholangitis,” Gastroenterology Review 16, no. 3 (2021): 184–187. 34584578 10.5114/pg.2021.108983 PMC8456759

[33] R. Fabia , R. Willén , A. Ar'Rajab , R. Andersson , B. Ahrén , and S. Bengmark , “Acetic Acid‐Induced Colitis in the Rat: A Reproducible Experimental Model for Acute Ulcerative Colitis,” European Surgical Research 24, no. 4 (1992): 211–225. 1505598 10.1159/000129209

[34] A. Bouhend , S. Keddari , I. Yahla , O. Sadouki , and M. Bououdina , “Therapeutic Benefits of Tuna Oil by In Vitro and In Vivo Studies Using a Rat Model of Acetic Acid‐Induced Ulcerative Colitis,” Applied Biochemistry and Biotechnology 196, no. 7 (2024): 3817–3843. 37787891 10.1007/s12010-023-04736-y

[35] H. M. Hafez , M. A. Ibrahim , W. Yehia Abdelzaher , A. A. Gad , S. Mohammed Naguib Abdel Hafez , and S. A. Abdel‐Gaber , “Protective Effect of Mirtazapine Against Acetic Acid‐Induced Ulcerative Colitis in Rats: Role of NLRP3 Inflammasome Pathway,” International Immunopharmacology 101, no. Pt A (2021): 108174. 34601335 10.1016/j.intimp.2021.108174

[36] D. K. Sahoo , R. M. Heilmann , B. Paital , et al., “Oxidative Stress, Hormones, and Effects of Natural Antioxidants on Intestinal Inflammation in Inflammatory Bowel Disease,” Frontiers in Endocrinology 14 (2023): 1217165. 37701897 10.3389/fendo.2023.1217165 PMC10493311

[37] M. S. Lokman , R. B. Kassab , F. A. M. Salem , et al., “Asiatic Acid Rescues Intestinal Tissue by Suppressing Molecular, Biochemical, and Histopathological Changes Associated With the Development of Ulcerative Colitis,” Bioscience Reports 44, no. 15 (2024): 00733, 10.14309/ctg.0000000000000733. PMC11130539 38699907

[38] M. H. El‐Saka , R. E. Abo El Gheit , A. El Saadany , G. M. Alghazaly , K. E. Marea , and N. M. Madi , “Effect of Spexin on Renal Dysfunction in Experimentally Obese Rats: Potential Mitigating Mechanisms via Galanin Receptor‐2,” Archives of Physiology and Biochemistry 129, no. 4 (2023): 933–942. 33632048 10.1080/13813455.2021.1887265

[39] W. Ou , H. Liu , C. Chen , et al., “Spexin Inhibits Excessive Autophagy‐Induced Ferroptosis to Alleviate Doxorubicin‐Induced Cardiotoxicity by Upregulating Beclin 1,” British Journal of Pharmacology 181, no. 21 (2024): 4195–4213. 38961632 10.1111/bph.16484

[40] M. M. Bahaa , S. K. Hegazy , M. M. Maher , M. M. Bahgat , and S. M. El‑Haggar , “Pentoxifylline in Patients With Ulcerative Colitis Treated With Mesalamine by Modulation of IL‐6/STAT3, ZO‐1, and S1P Pathways: A Randomized Controlled Double‐Blinded Study,” Inflammopharmacology 32, no. 5 (2024): 3247–3258. 39192162 10.1007/s10787-024-01560-6

[41] H. Ekoff , N. Rydell , P. M. Hellström , and R. Movérare , “Fecal and Serum Granulocyte Protein Levels in Inflammatory Bowel Disease and Irritable Bowel Syndrome and Their Relation to Disease Activity,” Clinical and Translational Gastroenterology 15, no. 10 (2024): e1. 10.14309/ctg.0000000000000733 PMC11500791 38920307

[42] S. Arabacı Tamer , K. S. Mermer , Ö. Erdoğan , et al., “Neuropeptide W Facilitates Chronic Gastric Ulcer Healing by the Regulation of Cyclooxygenase and NF‐κB Signaling Pathways,” Inflammopharmacology 32, no. 2 (2024): 1519–1529. 38227096 10.1007/s10787-023-01403-w PMC11006733

[43] B. Chami , N. J. J. Martin , J. M. Dennis , and P. K. Witting , “Myeloperoxidase in the Inflamed Colon: A Novel Target for Treating Inflammatory Bowel Disease,” Archives of Biochemistry and Biophysics 645 (2018): 61–71. 29548776 10.1016/j.abb.2018.03.012

[44] S. E. Gambaro , M. G. Zubiría , A. P. Giordano , et al., “Spexin Improves Adipose Tissue Inflammation and Macrophage Recruitment in Obese Mice,” Biochimica et Biophysica Acta (BBA) ‐ Molecular and Cell Biology of Lipids 1865, no. 7 (2020): 158700. 32201217 10.1016/j.bbalip.2020.158700

[45] R. M. Salama , S. F. Darwish , I. El Shaffei , et al., “Morus macroura Miq. Fruit Extract Protects Against Acetic Acid‐Induced Ulcerative Colitis in Rats: Novel Mechanistic Insights on Its Impact on miRNA‐223 and on the TNFα/NFκB/NLRP3 Inflammatory Axis,” Food and Chemical Toxicology 165 (2022): 113146. 35595039 10.1016/j.fct.2022.113146

[46] K. Nurmi , K. Silventoinen , S. Keskitalo , et al., “Truncating NFKB1 Variants Cause Combined NLRP3 Inflammasome Activation and Type I Interferon Signaling and Predispose to Necrotizing Fasciitis,” Cell Reports Medicine 5, no. 4 (2024): 101503. 38593810 10.1016/j.xcrm.2024.101503 PMC11031424

[47] R. E. Mostafa and R. F. Abdel‐Rahman , “Ezetimibe Alleviates Acetic Acid‐Induced Ulcerative Colitis in Rats: Targeting the Akt/NF‐κB/STAT3/CXCL10 Signaling Axis,” Journal of Pharmacy and Pharmacology 75, no. 4 (2023): 533–543. 36892981 10.1093/jpp/rgad013

[48] T. Olsen , R. Goll , G. Cui , et al., “Tissue Levels of Tumor Necrosis Factor‐Alpha Correlates With Grade of Inflammation in Untreated Ulcerative Colitis,” Scandinavian Journal of Gastroenterology 42, no. 11 (2007): 1312–1320. 17852866 10.1080/00365520701409035

[49] B. E. Sands and G. G. Kaplan , “The Role of TNFα in Ulcerative Colitis,” Journal of Clinical Pharmacology 47, no. 8 (2007): 930–941. 17567930 10.1177/0091270007301623

[50] M. Ranjbar Bushehri , N. Babaei , H. Esmaeili Gouvarchin Ghaleh , G. Khamisipour , and G. Farnoosh , “Anti‐Inflammatory Activity of Peiminine in Acetic Acid‐Induced Ulcerative Colitis Model,” Inflammopharmacology 32, no. 1 (2024): 657–665. 37855980 10.1007/s10787-023-01360-4

[51] M. A. El‐Rous , S. Saber , E. M. Raafat , and A. A. E. Ahmed , “Dapagliflozin, an SGLT2 Inhibitor, Ameliorates Acetic Acid‐Induced Colitis in Rats by Targeting NFκB/AMPK/NLRP3 Axis,” Inflammopharmacology 29, no. 4 (2021): 1169–1185. 34002329 10.1007/s10787-021-00818-7

[52] R. N. El Mahdy , M. A. Nader , M. G. Helal , S. E. Abu‐Risha , and M. E. Abdelmageed , “Eicosapentaenoic Acid Mitigates Ulcerative Colitis‐Induced by Acetic Acid Through Modulation of NF‐κB and TGF‐β/EGFR Signaling Pathways,” Life Sciences 327 (2023): 121820. 37263490 10.1016/j.lfs.2023.121820

[53] M. Shahid , M. Raish , A. Ahmad , et al., “Sinapic Acid Ameliorates Acetic Acid‐Induced Ulcerative Colitis in Rats by Suppressing Inflammation, Oxidative Stress, and Apoptosis,” Molecules 27, no. 13 (2022): 4139. 35807383 10.3390/molecules27134139 PMC9268465

[54] H. İ. Ceylan , Ö. Saygın , and Ü. Özel Türkcü , “Assessment of Acute Aerobic Exercise in the Morning Versus Evening on Asprosin, Spexin, lipocalin‐2, and Insulin Level in Overweight/Obese Versus Normal Weight Adult Men,” Chronobiology International 37, no. 8 (2020): 1252–1268. 32741294 10.1080/07420528.2020.1792482

[55] K. Abd el‐Fattah abul‐Fadle , N. El‐Huda A. Mohammed , R. M. Al‐Sayed , M. M. Abdul‐Rahman , and A. Ismael Farag , “Effect of Spexin Treatment on Cardiometabolic Changes in Obese Type 2 Diabetic Rats,” Al‐Azhar Medical Journal 49, no. 2 (2020): 735–758.

[56] E. K. Jo , J. K. Kim , D. M. Shin , and C. Sasakawa , “Molecular Mechanisms Regulating NLRP3 Inflammasome Activation,” Cellular & Molecular Immunology 13, no. 2 (2016): 148–159. 26549800 10.1038/cmi.2015.95 PMC4786634

[57] E. Latz , T. S. Xiao , and A. Stutz , “Activation and Regulation of the Inflammasomes,” Nature Reviews Immunology 13, no. 6 (2013): 397–411. 10.1038/nri3452 PMC3807999 23702978

[58] C. L. Chaudhary , P. Gurung , S. Jang , et al., “Synthesis, Activity and Mechanism of Alkoxy‐, Carbamato‐, Sulfonamido‐, Thioureido‐, and Ureido‐Derivatives of 2,4,5‐Trimethylpyridin‐3‐ol Against Inflammatory Bowel Disease,” Journal of Enzyme Inhibition and Medicinal Chemistry 35, no. 1 (2020): 1–20. 31619080 10.1080/14756366.2019.1677637 PMC6807866

[59] D. Zhang , H. Wan , R. Zhao , Y. Zhang , and H. Chen , “Eudragit S100 Coated Iron Oxide‐Chitosan Nanocomposites for Colon Targeting of 5‐Aminosalicylic Acid Ameliorate Ulcerative Colitis by Improving Intestinal Barrier Function and Inhibiting NLRP3 Inflammasome,” International Immunopharmacology 139 (2024): 112661. 39008936 10.1016/j.intimp.2024.112661

[60] C. Li , L. Deng , M. Pu , X. Ye , and Q. Lu , “Coptisine Alleviates Colitis Through Modulating Gut Microbiota and Inhibiting TXNIP/NLRP3 Inflammasome,” Journal of Ethnopharmacology 335 (2024): 118680. 39117021 10.1016/j.jep.2024.118680

[61] R. Zaharie , D. Valean , C. Popa , et al., “The Multifaceted Role and Regulation of Nlrp3 Inflammasome in Colitis‐Associated Colo‐Rectal Cancer: A Systematic Review,” International Journal of Molecular Sciences 24, no. 4 (2023): 3472. 36834883 10.3390/ijms24043472 PMC9959003

[62] S. Itani , T. Watanabe , Y. Nadatani , et al., “NLRP3 Inflammasome Has a Protective Effect Against Oxazolone‐Induced Colitis: A Possible Role in Ulcerative Colitis,” Scientific Reports 6, no. 1 (2016): 39075. 27966619 10.1038/srep39075 PMC5155456

[63] B. Yazgan , F. Avcı , G. Memi , and E. Tastekin , “Inflammatory Response and Matrix Metalloproteinases in Chronic Kidney Failure: Modulation by Adropin and Spexin,” Experimental Biology and Medicine 246, no. 17 (2021): 1917–1927. 34024143 10.1177/15353702211012417 PMC8424640

[64] G. Memi and B. Yazgan , “Adropin and Spexin Hormones Regulate the Systemic Inflammation in Adenine‐Induced Chronic Kidney Failure in Rat,” Chinese Journal of Physiology 64, no. 4 (2021): 194–201. 34472450 10.4103/cjp.cjp_13_21

