# Supplementary material for: Anti‐Inflammatory Effects of Spexin on Acetic Acid‑Induced Colitis in Rats via Modulating the NF‐κB/NLRP3 Inflammasome Pathway
Source: J Biochem Mol Toxicol. 2025 May 5;39(5):e70285. doi: 10.1002/jbt.70285 (PMC12050913; doi:10.1002/jbt.70285)
Supplement: Supplementary file 1 — suplm. Fig. 1.pdf. [file JBT-39-e70285-s001.pdf]

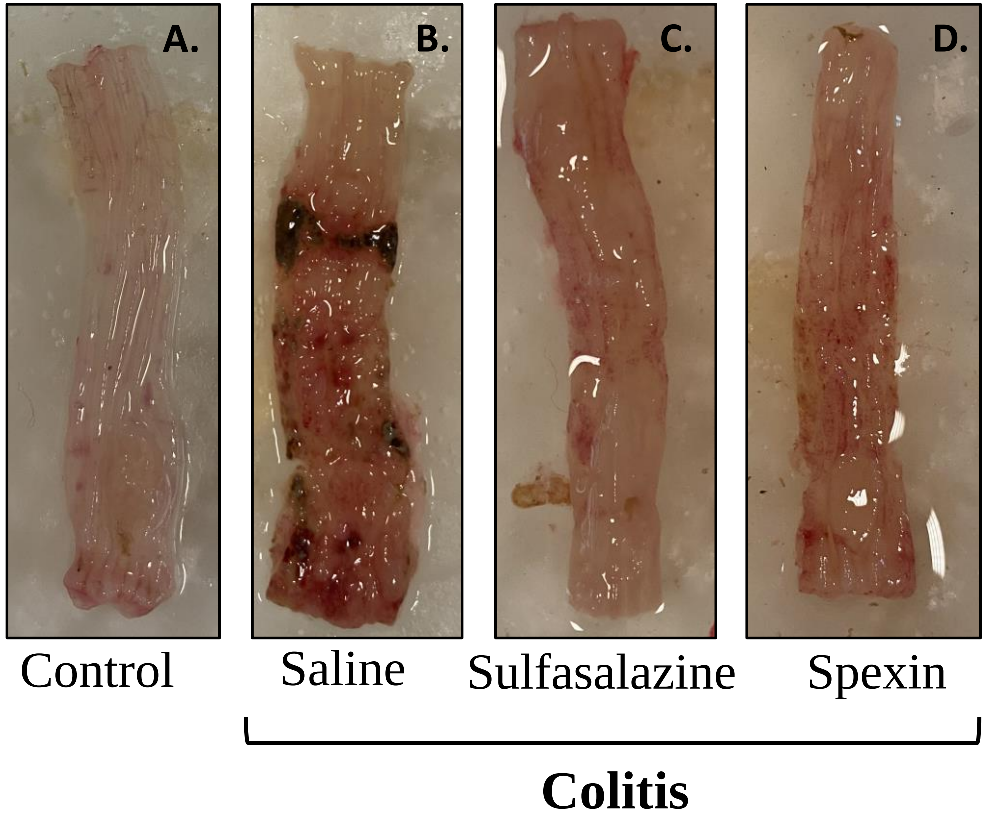

Supplementary Figure 1. Macroscopic presentation of acetic acid-induced colitis in rats. Normal colon treated with saline (A);acetic acid-applied colons with saline-treated (B) or sulfasalazine (C, 500 $mg.kg^{-1}$) or spexin (D, 50 $\mu g.\ kg^{-1}$). Colon tissue in control rat was normal (A). Saline-treated rats in the colitis group showed ulcerative areas in colon tissue, accompanied by considerable inflammation, ulceration, submucosal edema, and necrosis, whereas treatment with spexin or sulfasalazine markedly reduced these pathological damages.
